# Asthma Care in Resource-Poor Settings

**DOI:** 10.1097/WOX.0b013e318213598d

**Published:** 2011-04-15

**Authors:** Mario Sánchez-Borges, Arnaldo Capriles-Hulett, Fernan Caballero-Fonseca

**Affiliations:** 1 Allergy and Clinical Immunology Department, Centro Médico-Docente La Trinidad, Caracas, Venezuela

**Keywords:** asthma, asthma therapy, inhaled corticosteroids, leukotriene receptor antagonists, socioeconomic aspects

## Abstract

Asthma prevalence in low-to middle-income countries is at least the same or higher than in rich countries, but with increased severity. Lack of control in these settings is due to various factors such as low accessibility to effective medications, multiple and uncoordinated weak infrastructures of medical services for the management of chronic diseases such as asthma, poor compliance with prescribed therapy, lack of asthma education, and social and cultural factors. There is an urgent requirement for the implementation of better ways to treat asthma in underserved populations, enhancing the access to preventive medications and educational approaches with modern technological methods.

## Introduction

Asthma constitutes an important public health concern worldwide, affecting approximately 300 million people. Its prevalence has increased in recent years in many countries. The International Study of Asthma and Allergies in Childhood (ISAAC) demonstrated a world mean annual increase of 0.06% in adolescents and 0.13% in 6-to 7-year-old children between 1996 and 2003. In Latin America the increase was 0.32% in adolescents and 0.07% in children ages 6 to 7 [1]. International guidelines have defined goals for asthma control through a correct diagnosis, assessment of severity, adequate therapies, and patient education [2]. They include control of symptoms, prevention of exacerbations, reduction of visits to the emergency department and hospitalizations, minimal utilization of reliever medications, no limitations of physical activity, normalization with absence of diurnal variations of lung function, and lack of adverse effects from asthma medications.

Recent studies carried out in various regions of the world concluded that, despite guidelines presently available, asthma control is not obtained in most patients [3-5]. As an example, the AIRLA Study (Asthma Insights and Reality in Latin America) demonstrated that between 5 and 15% of patients had severe symptoms and between 40 and 77% needed hospital medical assistance [5].

These investigations concluded that there is a poor standard of care in all regions, with resource-poor countries faring no worse than rich countries in failing to achieve the GINA goals, and both patients and care providers underestimated asthma severity and a significant proportion of patients and care providers paid little attention to use of inhaled corticosteroids. Among recommendations for better outcomes, experts suggested improving the access and afford-ability of corticosteroids, increasing patient and physician education, and implementing social, cultural, and politically relevant management plans.

It is clear that asthma is a disease with great impact because of its high prevalence, the compromise of patient's quality of life, and its direct and indirect costs. The main direct costs of asthma are associated with lack of control, with frequent exacerbations and hospitalizations, visits to the emergency services, and nonprogrammed ambulatory physician consultations.

The purpose of this paper is to describe the influences of socioeconomic factors on the clinical expression and management of asthma with special consideration to the status of asthma care in Latin America.

## Difficulties in asthma care

Asthma is a complex and highly prevalent disease. It has been recognized that management of asthma is a difficult task. Some obstacles to good disease control are the lack of education about asthma, the absence of effective prevention, multiple problems with delivery of drugs through the inhalation route, and the unavoidable fact that treatment guidelines are too complicated. Additionally, the majority of asthmatic patients do not have access to effective care. This is even worse because of cultural barriers, the low priority given to asthma by health authorities, and costs of medications [6].

Many preventable risk factors for asthma have been identified, including tobacco smoke, intradomiciliary air contamination, allergens, and occupational agents. Asthma and its risk factors generally receive insufficient attention from the health community, patients, families, and the media. Consequently, there is a lack of recognition, underdiagnosis, undertreatment, and insufficient prevention.

## Deprivation and asthma

The high prevalence of asthma implies various additional problems such as the limitations related to medical attention and availability of basic medications in countries with limited economic resources, the decrease in quality of life of patients and their families, the increased use of public health services, the high costs of health management, the increased school and work absenteeism and attendance, and the increase of mortality rates [7]. It has been estimated that asthma is responsible for 250,000 deaths every year, and the mortality is higher in median- and low-income countries [8].

**Figure 1:**
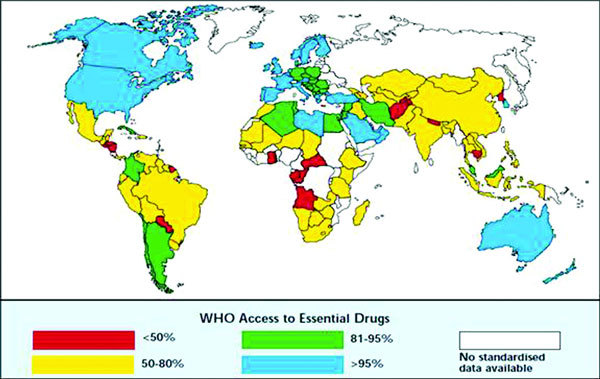
**World map of the proportion of the population with access to essential drugs**. From http://www.ginasthma.org. Accessed January 20, 2011.

The difficulties mentioned above are worse in deprived populations. For example, the adherence to asthma medication in poor populations is lower than 50%, a figure observed in studies from different nondeprived European and North American countries, whereas the access to essential drugs for adequate asthma treatment, especially to inhaled corticosteroids, is quite low in many developing countries (Figure 1). A study carried out in Porto Alegre, Brazil, found that the overall rate of compliance in people with asthma was 51.9% [9]. In addition, the study by Watson and Lewis concluded that many patients with asthma living in developing countries are not receiving adequate therapy because the required drugs are not available in their area or are prohibitively expensive [10].

In a study done in 24 developing countries in Africa and Asia, Edwards observed that oxygen was available in 50% of the clinics, electricity in 25 of 41 centers, peak flow meters only in 3 sites or 26 of 41 doctors, rapid-acting bronchodilators in all, inhaled corticosteroids in 50% (considered too expensive), and no offering of patient education [11].

According to Rona, deprivation is consistently associated with increased severity of asthma but not with a higher prevalence [12]. This has also been shown by Baker et al in a study that demonstrated increased severity in patients who live in rented houses and lower severity in patients seen in private institutions [13].

Furthermore, it has been observed that guidelines adopt a general (not individual) approach to disease management, and that in poor countries the commitment of public health officials is low and there is a large diversity of uncoordinated health systems, variations in availability of medications, and limitations in resources. Additionally, in many primary care settings there is resistance to the implementation of National or International Guidelines for asthma diagnosis and management.

Poverty has other effects on asthma. It contributes to exacerbations, is a determinant of the quality of care that patients receive, and determines the psychosocial behavior which in turn impacts the management and prognosis of the condition. The lack of access to essential basic therapy, mainly inhaled steroids, is especially notorious in resource-poor countries.

Because of cost concerns, in deprived areas inhaled steroids, long-acting beta agonists, and other medications are not available universally, and new drugs, new devices, and formulations are too expensive.

Some investigators have proposed that in such settings the use of older steroids and cheaper oral drugs such as theophylline, oral corticosteroids, and oral bronchodilators would be a plausible alternative to no treatment [14]. Leukotriene receptor antagonists, having better adherence because of their oral administration, would also be useful if costs can be reduced and made accessible to a larger number of persons with asthma living in poor areas [15-21], although it is known that leukotriene modifiers are in general less effective than inhaled corticosteroids for the reduction of asthma morbidity and mortality. Increased educational efforts, including nonphysician asthma educators, are required, in addition to providing global access to core medications at affordable prices and encouraging better health care provider and patient education.

## Cultural and socioeconomic factors

In a study carried out in East London, South Asians showed less confidence in controlling their asthma, were unfamiliar with preventive medications, and often expressed less confidence in their general practitioners. They managed asthma exacerbations with family advocacy, without systematic changes in prophylaxis, and without use of systemic corticosteroids. In general, they attended medical practices with weak strategies for asthma care and consequently showed increased risk of hospital admission [22]. Authors postulated that this could reflect either an intrinsic cultural characteristic or the difficulties of coping with asthma in deprived circumstances where racism is common and health services are often inadequate and inappropriate.

It is generally accepted that good access to primary care is associated with reduced risk of hospitalization. A behavioral intervention for doctors that promotes a partnership style of medical practice would increase patient's confidence and reduce their use of health services.

Another investigation by Moudgil and Honeybourne on differences in asthma management between white European and Indian subcontinent ethnic groups living in socioeconomically deprived areas in Birmingham, UK, concluded that the management of both ethnic groups centered on drug prescription, delivery techniques, and compliance, but was deficient, particularly in the Indian subcontinent group, in patient's understanding of the disease and self-management [23].

## Asthma is a public health problem in Latin America

Asthma constitutes an important sanitary issue not only in Latin American countries but also in Hispanic people living in North America [24]. Cooper et al suggested that the increased prevalence of asthma observed in some countries in Latinoamerica such as Brazil and Costa Rica is associated with underprivileged populations living in cities, whereas the disease remains relatively rare in many rural populations. These authors suggested that causes of asthma in Latin America are likely to be associated with urbanization, migration, and the adoption of a modern "Westernized" lifestyle and environmental changes that follow these processes [25]. When hospitalizations due to asthma in Latin America are analyzed, it can be seen that one of the factors involved is low utilization of controller medications, especially inhaled steroids [26]. The increased use of inhaled corticosteroids and possibly the reduction of theophylline therapy were suggested as the most relevant factors accompanying the decrease of asthma mortality in Argentina [27].

We will discuss the present situation of asthma in Venezuela as an example of the difficulties for asthma control in many Latin American settings. Health statistics confirm that asthma constitutes the second cause, after viral syndromes, for consultation in the outpatient clinics of the Ministry of Health and Social Development of Venezuela with 865,738 visits in the year 2000, most likely an underestimation. It is the first among respiratory diseases, above tonsillitis, rhinopharyngitis, acute bronchitis, and pharyngitis.

Asthma treatment is focused mainly on exacerbations, and therefore asthma is a major burden in emergency department visits and hospitalizations in this country. Eighty-six percent of all medications sold for the treatment of asthma are bronchodilators and other rescue drugs.

In addition to this, in a study performed by Proyecto Venezuela (Fundacredesa) looking into growth and development data across the country, an increased frequency of asthma complaints called investigator's attention. When socioeconomic levels of the patients were analyzed using a questionnaire similar to ISAAC's study, it was observed that there was a significantly increased prevalence of asthma in individuals from lower socioeconomic class (levels IV and V) as compared with higher class (levels I to III) (Table 1). Authors concluded that in this population asthma affects mainly individuals from low socioeconomic levels [28]. These investigations have suggested that asthma in Venezuela is a disease of young, urban, and poor people. It is likely that the same is occurring in many other large cities in Latin America.

**Table 1:** Asthma As a Disease of the Poor

	< 2 Years **(*n ***= **11060)**		2-6.99 Years **(*n ***= **10698)**		7-13.99 Years **(*n ***= **9016)**		14-19.99 Years **(*n ***= **8716)**	
	
Social level	*n*	%	*n*	%	*n*	%	*n*	%
Total	661	6	1457	13.6	1520	16.9	1209	13.8
I + II = III	28	4	89	6.1	350	23.0	227	18.7
IV*	226	*34*	378	*25.9*	446	*29.4*	433	*35.8*
V*	407	*62*	990	*68.0*	724	*47.6*	549	*45.5*

Proyecto Venezuela, División de Investigación sobre la Familia, 1981-1987.

*P < 0.05.

The last revision of the National Asthma Program, based on GINA guidelines, was done in 1998. According to the World Health Organization the impact from asthma on public health budgets depends on emergency department visits and hospitalizations, and direct and indirect costs show a 1:1 rate; asthma requires about 1-2% of the total budget of the Ministry of Health. In Venezuela in 2006 this amounted to U.S. $120 per capita (for asthma $67 million per year) [29].

Also according to the World Health Organization ambulatory services health costs should be U.S. $46.34 per patient, although this amount may not be applicable to countries with different standards of living. It means that for 1 million of acute asthma visits per year $46.34 million are required. If a 10% hospitalization rate per year is assumed, with a 4-day mean duration and $81.83 per day cost, $32.73 million for admitted asthmatics are needed. In total, the cost of asthma in Venezuela per year reaches the figure of $78.34 million.

Recent studies have shown a reduction in the number of hospitalizations caused by asthma in various countries when effective preventive and controller measures are implemented [30]. Educational approaches for physicians and patients are essential to improve asthma control.

Brazil is a country that has recently taken the leadership in Latin America in regard to programs for asthma education and management. Lasmar et al reported on the Wheezy Child Program, the experience of the Belo Horizonte Pediatric Asthma Management Program. Using educational strategies, they observed a reduction of hospitalization rates for asthma and pneumonia in children, from 40.5% before admission to 8.6% after being included in the program [31]. Favorable results were also obtained in patients with severe asthma in Salvador, Bahia, as reported by Souza-Machado et al [32].

## Interventions to improve Asthma care in deprived populations

Experts recognize that educational interventions are crucial to improve asthma care in underserved populations. De Oliveira et al compared asthma outcomes in patients receiving or lacking education for asthma. Although no differences in lung function, as determined by pre- and postbronchodilator peak flow rates, were present, significant improvements in the educational group for emergency room visits, nocturnal symptoms, symptom frequency, and quality of life were observed. Patients in the treated group showed adequate use of metered dose inhalers, better knowledge of rescue and preventive medications, and improved environmental control. In regard to medications, the educational group showed an increased frequency of inhaled corticosteroid use after implementation of the program [33].

Key educational issues for the patient include the understanding of the role of inflammation in asthma, how preventer/controller and reliever medications work and when they should be used, what to do in an emergency situation (with self-management instructions), and adequate education on the proper technique for the use of inhaled medications.

## Conclusions

Asthma prevalence in deprived regions is high and shows increased severity. Reasons for inadequate asthma control in poor populations include low accessibility to effective controller medications, weak infrastructure of health services for the management of chronic diseases, poor adherence to the therapy, lack of educational approaches, and social, cultural, and language barriers. There is a need for the implementation of improved ways to treat asthma in these populations, enhancing the access to preventive medications and to educational interventions which include modern technological tools.
